# The Multifaceted Role of Neuroprotectin D1: Physiological, Pathophysiological, and Pharmacological Insights in Neurodegenerative Diseases

**DOI:** 10.2174/011570159X365720250225080257

**Published:** 2025-04-07

**Authors:** Bushra Zia, Mariam Elmeky, Sheikh Azimullah, Niraj Kumar Jha, Mohamed Fizur Nagoor Meeran, Shreesh K. Ojha

**Affiliations:** 1 Department of Pharmacology and Therapeutics, College of Medicine and Health Sciences, United Arab Emirates University, PO Box: 15551, Al Ain, UAE;; 2 School of Bioengineering & Biosciences, Lovely Professional University, Phagwara, 144411, Punjab, India;; 3 Zayed Center for Health Sciences, College of Medicine and Health Sciences, United Arab Emirates University, PO Box: 15551, Al Ain, UAE

**Keywords:** Cyclooxygenases, docosahexaenoic acid, inflammatory cytokines, lipoxygenases, Neuroprotectin D1, neuroprotective, neurodegenerative diseases, protectin D, pro-resolving lipid mediators

## Abstract

Neuroprotectin D1 (NPD1) has emerged as an integral lipid mediator with significant implications for maintaining neurological health. Being derived from docosahexaenoic acid (DHA), NPD1 is a specialized pro-resolving lipid mediator (SPM), consisting of a unique structure that attributes potent anti-inflammatory and neuroprotective properties crucial for maintaining nervous system homeostasis. It exerts its actions through diverse mechanisms, including the inhibition of proinflammatory cytokines, modulation of apoptosis, and promotion of cellular survival pathways. The dysregulation or deficiency of NPD1 has been implicated in the onset and progression of several neurodegenerative diseases, such as Alzheimer’s, Parkinson’s, and stroke, underscoring its critical role in maintaining neuronal health and disease prevention. Abnormal NPD1 signalling is associated with neuroinflammation, oxidative stress, and neuronal apoptosis, which in turn contribute significantly to the progression of neurological disorders. Understanding these pathways offers insights into potential therapeutic strategies aimed at targeting NPD1 to mitigate neurodegenerative processes and facilitate neural repair. The efforts in developing NPD1 analogs or mimetics are focused on enhancing endogenous NPD1 levels, attenuating neuroinflammation, and preserving neuronal integrity in disease contexts. This review provides a comprehensive exploration of NPD1, encompassing its structural characteristics, biochemical pathways, physiological roles, pathophysiological implications, and potential therapeutic applications in neurological disorders. Further, research into elucidating the precise mechanisms of NPD1 reveals that its clinical efficacy is crucial for harnessing its full potential as a therapeutic tool for neuroprotection and neural repair.

## 1 INTRODUCTION

Neuroprotectin D1 (NPD1) belongs to a class of specialized pro-resolving lipid mediators (SPMs) derived from the omega-3 essential fatty acid docosahexaenoic acid (DHA) through a series of enzymatic reactions catalyzed by lipoxygenases [1]. SPMs play a critical role in restoring neuronal homeostasis by resolving inflammation associated with neuronal injury, including protein misfolding and resulting proteotoxicity [2]. Whereas earlier studies primarily focused on inhibiting the synthesis of inflammatory substances, recent research has pivoted towards actively resolving inflammation. Proinflammatory lipid mediators, including the prostaglandins (PGs) and thromboxanes (TXs), are produced from arachidonic acid *via* the enzymes cyclooxygenases (COXs), whereas the leukotrienes are synthesized *via* the LOXs. These mediators are involved in typical inflammatory responses inducing fever and pain. Although inflammation serves as a protective response to clear causative agents and consequences of tissue injury, chronic inflammation eventually leads to tissue damage and loss of function. Uncontrolled inflammation has profound implications in exacerbating the pathogenesis of several diseases, including cerebrovascular and cardiovascular diseases, Alzheimer’s disease (AD), cancer, and obesity [3].

Proinflammatory mediators are crucial signaling molecules that regulate inflammation due to their small molecular weights, localized action, and activation pathways [4]. The migration, recruitment, and adhesion of leukocytes at the site of inflammation are orchestrated by local vascular dynamics, including blood flow regulation, vascular capillary dilation, and permeability, which are intricately controlled by proinflammatory PGs and leukotrienes (LTs) [4, 5]. Cyclooxygenases and LOXs catalyze the formation of these mediators activated by tissue injury or microbial invasion [6]. Prostaglandins synthesized from arachidonic acid not only initiate cellular response relevant to inflammation but also signal the end of inflammation by activating the transcription of LOXs involved in the production of lipoxins as the inflammation resolves. SPMs are generally metabolites of the omega-3 polyunsaturated fatty acids (ω-3 PUFA) like DHA and eicosapentaenoic acid (EPA) and include maresin 1 (MaR1), resolvin D1(RvD1) and NPD1. An exception is lipoxin-A4 (LXA4), derived from the omega-6 polyunsaturated fatty acid (ω-6 PUFA), which is produced from arachidonic acid by the enzymatic action of LOXs including 15-lipoxygenase-1 (15-LOX-1) and 5-lipoxygenase-1 (5-LOX-1). The synthesis of protectins, resolvins, and lipoxins, derived from omega-3 and omega-6 fatty acids through lipoxygenase (LOX) enzymes, is detailed in Fig. (**1**). Lipoxins (LXs) were the first identified pro-resolving mediators *in vivo*, produced *via* eicosanoid class switching of prostaglandin (PGs) and leukotriene (LTs) to lipoxins (LX) during the later phases of inflammation. This switch leads polymorphonuclear neutrophils (PMNs) to shift from producing leukotriene B4 to producing lipoxin, thus facilitating the resolution of inflammation [5].

Numerous studies have shown that PMNs undergo class switching based on environmental cues [3, 5, 7, 8]. Lipoxins (LXs) facilitate resolution by inhibiting PMN infiltration, reducing vascular permeability, and promoting macrophage-mediated clearance of neutrophils. This anti-inflammatory effect is distinct from pro-resolution mechanisms. The synthesis of NPD1 from DHA, which is enriched in the central nervous system and incorporated into membrane phospholipids, is tightly regulated by phospholipase A2 (PLA2) [9]. DHA is incorporated into membrane phospholipids during the synthesis of docosahexaenoyl-coenzyme A and released from the membrane in a hydrolysis reaction catalyzed by PLA_2_ [10]. DHA can be derived from dietary intake or through elongation and desaturation of α-linolenic acid in hepatocytes, packaged into lipoproteins, and delivered to the brain and other tissues [11]. In contrast, omega-6 polyunsaturated fatty acids (PUFAs) such as arachidonic acid are synthesized more efficiently from linoleic acid, highlighting the need for omega-3 PUFAs dietary supplements.

Serhan *et al*. in 2004 discovered an endogenous molecule known as 10,17-docosatriene (DT), which was named NPD1, due to its notable neuroprotective effects observed in models of brain ischemia-reperfusion and oxidative stress in retinal pigment epithelial (RPE) cells [2, 12]. Neuroprotectin D1 was named for its potent ability to counteract pro-apoptotic signaling and its designation as the first identified neuroprotective mediator derived from docosahexaenoic acid (DHA). Further, Serhan expanded the understanding of NPD1’s role, revealing that its biological effects were not limited to the nervous system. Consequently, NPD1 was reclassified as protectin D1 (PD1) in the year 2005 [13]. By 2006, the chemical structure of PD1 was thoroughly elucidated by correlating biological samples with stereochemically pure and enantio-enriched isomers obtained through total synthesis [14].

## 2 STRUCTURAL CHEMISTRY, BIOSYNTHESIS, AND FUNCTIONAL MECHANISMS OF NEUROPROTECTIN D1

Protectin D1, a polyunsaturated fatty acid with two hydroxyl groups at the C22 position, is synthesized from all-Z-docosahexaenoic acid and belongs to a recently recognized class of endogenous molecules called specialized pro-resolving lipid mediators [15]. Several tissues, such as the retinal pigment epithelial cells, lung epithelial cells, peripheral blood mononuclear cells (PBMC), and neural tissues, produce PD1 in response to inflammatory signals. Research indicates that in PBMCs, endogenous docosahexaenoic acid (DHA), the precursor to PD1, is released through the action of phospholipase A2 [13, 16]. Within PBMCs, PD1 synthesis is notably elevated in cells exhibiting a Type 2 T-helper cell (Th2) phenotype, suggesting that T-cell differentiation significantly influences PD1 biosynthesis. The presence of interleukin 4 (IL-4), a potent inflammatory mediator, drives the differentiation of PBMCs into Th2 lymphocytes. Additionally, activated Th2 cells release more IL-4, which in turn upregulates the enzyme 15-LOX-1. This enzyme, a non-heme iron dioxygenase, introduces oxygen into free and esterified ω-3 polyunsaturated fatty acids like DHA in a stereospecific manner.

The biosynthesis of PD1 involves three key steps, with 15-LOX-1 being pivotal in each step, as has been depicted in Fig. (**2**). Initially, 15-LOX-1 acts on DHA to produce a (17S)-hydro(peroxy)-DHA intermediate. This intermediate is then converted into a 16(17)-epoxide-containing molecule. The final step involves the enzymatic hydrolysis of this epoxide intermediate, leading to the formation of PD1 [13]. DHA is a relatively effective substrate for LOXs, leading to the formation of various hydroxyldocosahexaenoic acids (HDHA). Specifically, 4- and 7-HDHA are produced *via* 5-LOX [17], while 11- and 14-HDHA result from 12-/ω9-LOX activity [17, 18], and 17-HDHA is generated through 15-/ ω6-LOX [19]. Beyond these mono-oxygenation products, DHA can also be double-oxygenated by 15-/ω6-LOX to produce protectin-DX (PDX). PDX features a distinct conjugated triene geometry compared to NPD1 has been elaborated elsewhere [20].

Lipoxygenases are non-heme iron-containing enzymes, a single polypeptide with a molecular weight of around 75-80 kD in animals. The iron atom in the enzyme is generally present in the ferrous state. Upon activation, the ferrous state oxidizes to the ferric state, which is involved in the catalysis of the substrate, usually essential fatty acids containing cis-double bonds like PUFAs. The LOX enzymes (5-LOX, 12-LOX, and 15-LOX) oxygenate PUFAs that contain one or more Z, Z-1,4-pentadiene moieties [21]. The naming of LOX isoenzymes is determined by the position of the carbon atom, from the tail, in the substrate that is oxidized [22]. These enzymes operate by removing a hydrogen atom from a cis, cis-1,4-pentadiene structure within a polyunsaturated fatty acid (PUFA), followed by the insertion of oxygen to form a hydroperoxide. The location of oxygen insertion is influenced by the orientation of the substrate entering the enzyme's active site first. For instance, human reticulocyte 15-lipoxygenase-1 [h15-LOX-1 (ALOX15)] predominantly produces the ω-6 hydroperoxide, 15S-hydroperoxy-5Z,8Z, 11Z,13E-eicosatetraenoic acid (15S-HpETE) from arachidonic acid, and 17S-hydroperoxy-4Z,7Z,10Z,13Z,15E,19Z-docosahexaenoic acid (17S-HpDHA) from DHA, as the fatty acid substrate enters the active site from its methyl end.

Biosynthetic pathway studies demonstrate that distinct families of SPMs are formed during the resolution phase of acute inflammation [12, 23]. In 2002, Serhan *et al*. reported the synthesis, isolation, and characterization of various metabolites derived from the oxygenation of DHA, including NPD1, with a peculiar E, E, and Z-conjugated triene geometry [24]. The biosynthesis of NPD1 involves both LOX-mediated oxygenation and dehydration reactions. Initially, a LOX enzyme abstracts a hydrogen atom from the C12 position of 17S-hydroperoxy-docosahexaenoic acid (17S-HpDHA), leading to the formation of a hydroperoxide intermediate. This intermediate then undergoes dehydration to produce 16S,17S-epoxyDHA, which is subsequently hydrolysed to yield NPD1 [14]. The first step in this biosynthetic pathway involves the formation of 17(S)-HpDHA through the stereoselective insertion of molecular oxygen at C17, following an antarafacial hydrogen abstraction at C15 of DHA. These processes occur with high stereoselectivity, favoring the S-configuration at C17. The presence of 15-LOX enables the antarafacial abstraction of hydrogen at C15 and the addition of oxygen at C17 to happen simultaneously, thereby ensuring the S-chirality at C17 [21]. This intermediate peroxyl radical is then reduced to form 17S-HpDHA. This compound has been identified in various biological contexts, including human blood, leukocytes, glial cells, and mouse brain [25].

The stable E, Z-diene structure produced is more thermodynamically favourable compared to the Z, Z-diene found in other PUFAs [21]. In the next stage, the reactive 17S-HpDHA undergoes a second hydrogen abstraction at C12, followed by an intramolecular oxygen attack at C16, leading to the formation of the 16S,17S-epoxyprotectin intermediate through water loss. These intermediate features a conjugated E, Z-diene structure at C10-C13 [26]. Specifically, h15-LOX-1 generates 17S-HpDHA, which then loses a hydrogen atom from the ω-11 carbon (or C12) to form the crucial intermediate, 16S,17S-epoxy-4Z,7Z,10Z,12E,14E,19Z-docosa-hexaenoic acid (16S,17S-epoxyDHA), possessing a UV-active triene with 10Z,12E,14E geometry. This intermediate then undergoes a stereoselective nucleophilic water addition at C10, catalysed by an unidentified hydrolase, which converts the triene geometry from 10Z,12E,14E to 11E,13E,15Z in PD1. The chiral centre at C-10 is formed with the R configuration, as confirmed by comparisons with synthetic PD1 material, whereas the S-configuration at C17 is retained [27].

In addition to h15-LOX-1, both h12-LOX and human epithelial 15-LOX-2 (h15-LOX-2) are potential biocatalysts for NPD1 production due to their reactivity with DHA. While h12-LOX mainly produces the ω-9 product, 14S-HpDHA, it also generates the ω-6 side product, 17S-HpDHA [28]. In addition to h15-LOX-1, both h12-LOX and human epithelial 15-LOX-2 (h15-LOX-2) are potential biocatalysts for NPD1 production due to their reactivity with DHA. While h12-LOX mainly produces the ω-9 product, 14S-HpDHA, it also generates the ω-6 side product, 17S-HpDHA. Similarly, h15-LOX-2 exhibits a reaction specificity akin to h15-LOX-1, with 17S-HpDHA as the predominant product, though the dehydration step to form 16S,17S-epoxyDHA has not been reported for these enzymes [29].

Protectin DX (PDX) is produced by soybean LOX through the double oxygenation of DHA, followed by reduction to form the dialcohol [30]. Conversely, the proposed biosynthetic route for PDX involves the oxygenation of 17S-HpDHA (or 17-HDHA) at C10 by a LOX isozyme, with subsequent reduction by glutathione peroxidase, GPx-4 [30]. Given the potent biological actions of NPD1 and PDX, it is of significant interest to investigate the possible *in vitro* biosynthetic pathways for both molecules to propose possible *in vivo* biosynthetic routes for NPD1 and PDX biosynthesis [31].

Neuroprotectin D1 derived from DHA is a specialized lipid mediator crucial for resolving inflammation and protecting neurons in the central nervous system (CNS). It helps maintain tissue balance and supports neuronal survival during inflammation without compromising immune function. The role of NPD1 includes regulating neuroinflammation, preserving neuronal health, and modulating pain pathways in the CNS. These properties make NPD1 a promising mediator for treating neurodegenerative diseases, acute neurological injuries, and chronic pain syndromes by leveraging its anti-inflammatory and neuroprotective effects [2, 32]. NPD1 suppresses the expression and release of proinflammatory cytokines such as interleukin-1β (IL-1β), tumor necrosis factor-alpha (TNF-α), and interleukin-6 (IL-6) from microglia and astrocytes [4, 32]. These cytokines are key mediators of neuroinflammatory responses and contribute to neuronal damage in various neurological disorders [33-36].

Mechanistically, NPD1 suppresses the activation of nuclear factor-kappa B (NF-κB), a transcription factor crucial for the expression of proinflammatory genes [4], by upregulating the expression of c-Rel, which is involved in neuroprotection [37]. Beyond the aforementioned factors, the NF-κB also plays an intricate role in regulating certain microRNAs involved in exerting its neurotoxic effect in AD. NF-κB regulates the expression of certain microRNAs such as miRNA-125b, miRNA-9, miRNA-155, miRNA-34a, and miRNA-146a [38], among which, the expression of miRNA-125b is highly upregulated in AD tissues and is found in significantly higher concentrations in the brain. In a comparable manner, NF-κB-induced mir-125b has been demonstrated to inhibit the expression of 15-LOX, an enzyme crucial for converting DHA into NPD1 and activating the vitamin D3 receptor (VDR). The damage to the brain caused by reactive oxygen and reactive nitrogen species (ROS and RNS) is mitigated by the protective functions of NPD1 and VDR [39]. By attenuating the amyloidogenic pathway of amyloid-beta (Aβ42) synthesis, NPD1 attenuates the inflammatory cascade and promotes the resolution of neuroinflammation.

Neuroprotectin D1 bioactivity may be elicited through a putative receptor and, in turn, upregulate the expression of E3 ubiquitin ligases such as Baculoviral IAP repeat Containing 2 (BIRC2) and Baculoviral IAP repeat Containing 3 (BIRC3), involved in sequestering factors responsible for apoptosis [40-42] and other transcription factors, and as a consequence, decreases proinflammatory gene expression [43]. Uncompensated oxidative stress triggers the activation of calcium ionophore A23187, or to a lesser extent, IL-1β, leading to the synthesis of NPD1 while simultaneously increasing the endogenous free DHA pool to levels three to four times higher than the amount of NPD1 synthesized [2]. NF-κB regulates a myriad of cellular factors that are involved in the progression of neuro-inflammatory pathways leading to neurodegeneration in neuronal cells, making it crucial to understand how NPD1 mitigates the impact of NF-κB [44]. Microglia, the resident immune cells of the CNS, play a dual role in neuroinflammation by exerting both neuroprotective and neurotoxic effects.

Neuroprotectin D1 promotes the shift of microglia towards an anti-inflammatory phenotype, characterized by reduced production of ROS and inflammatory mediators. Microglia, as resident macrophages in the CNS, are crucial for immune surveillance and respond to various pathological stimuli [45]. Upon activation, they adopt a proinflammatory phenotype, characterized by a pronounced ‘amboeid’ morphology and increased production of proinflammatory molecules like IL-1α, TNF-α, and C1q [46, 47]. The phenotypic transition of healthy astrocytes to the harmful A1 type, triggered by cytokines released from activated microglia, results in a loss of their supportive functions and promotes neuronal death. Consequently, A1 astrocytes fail to preserve synaptic functions and phagocytic activity while concurrently releasing neurotoxins that cause neuronal death [47, 48]. Neuroprotectin D1 counteracts these morphological changes by restoring microglia and astrocytes in the hippocampus and prefrontal cortex to their original states, thereby reducing inflammation and modulating cytokine secretion in a mouse model [49]. Additionally, NPD1 enhances the phagocytic activity of microglia, facilitating the clearance of cellular debris and apoptotic cells-a critical step in inflammation resolution and tissue repair. Neuroprotectin D1 accelerates the clearance of inflammatory infiltrates from the CNS by enhancing macrophage-mediated phagocytosis [50]. This includes the removal of apoptotic neutrophils and other immune cells, thereby reducing inflammation duration and promoting tissue repair.

Neuroprotectin D1 inhibits proinflammatory signaling related to macrophage/neutrophil infiltration, enhances macrophage phagocytosis towards apoptotic cells, and accelerates tissue repair [51]. Furthermore, NPD1 reduces vascular permeability and stabilizes the blood-brain barrier (BBB), preventing the extravasation of immune cells and inflammatory mediators into the brain by increasing the expression of tight junction (TJ)-associated proteins such as Zonula occludens- 1(ZO-1), claudin-5, and occludin [52]. By maintaining BBB integrity, NPD1 limits the spread of inflammation and protects neighbouring healthy tissues from damage. Lipid mediators like PGs and leukotrienes (LTs) are strong inflammatory signaling molecules that can worsen neuroinflammation. COX-2, an inducible enzyme triggered by IL-1β, plays a key role in producing prostaglandins from arachidonic acid. However, NPD1 mitigates the production of these proinflammatory mediators by reducing the expression of COX-2 [2].

Neuroprotectin D1 inhibits the enzymatic pathways responsible for the synthesis of PGs and LTs, thereby reducing their production and attenuating their proinflammatory effects within the CNS. Apoptosis, or programmed cell death, contributes to neuronal loss in neurodegenerative disorders such as AD and Parkinson's disease (PD). Neuroprotectin D1 enhances the expression of anti-apoptotic proteins while suppressing the activation of pro-apoptotic pathways within neurons [49, 53]. By maintaining mitochondrial function and regulating B-cell lymphoma 2 (Bcl-2) family proteins, NPD1 promotes neuronal survival and preserves functional integrity in the face of cytotoxic insults.

Neuroprotectin D1 counteracts this pro-apoptotic activity by promoting differential expression of Bcl-2 family proteins, up-regulating anti-apoptotic Bcl-2, Bcl-xL, and Bcl-2 related protein A1(Bfl-1/A1), and by attenuating the expression of pro-apoptotic Bax, Bad, and Bid. Oxidative stress, characterized by an imbalance between ROS production and antioxidant defences, plays a pivotal role in neurodegeneration. Furthermore, NPD1 reduces the activation of caspase-3 induced by oxidative and proteotoxic stress [2].

Moreover, NPD1 enhances cell survival under uncompensated oxidative stress by significantly increasing the expression of ring finger protein 146 (Iduna). This upregulation of Iduna offers substantial protection to cells facing uncompensated oxidative stress by facilitating DNA repair [54, 55]. This antioxidant defense system reduces oxidative damage to neuronal membranes, proteins, and DNA, thereby mitigating neurotoxicity and preserving neuronal viability. Additionally, Specialized Pro-resolving Mediators (SPMs) may alleviate excessive nociceptor sensitization by inhibiting specific channels such as transient receptor potential ankyrin 1 (TRPA1) and transient receptor potential vanilloid 1 (TRPV1), thereby aiding in pain resolution [56]. Both MaR1 and NPD1 can reduce neuroinflammatory pain by suppressing TRPV1 activity in dorsal root ganglion (DRG) neurons through the inhibition of PKA and ERK pathways [50, 57]. NPD1 contributes to pain relief and improves pain management strategies in neurological disorders. Pain sensation is often exacerbated by neuroinflammation, where proinflammatory cytokines and lipid mediators sensitize nociceptive neurons. NPD1's anti-inflammatory actions, including the inhibition of cytokine production and leukocyte infiltration, attenuate peripheral sensitization and neurogenic inflammation. This dual modulation of nociceptive receptors and neuroinflammatory pathways highlights the potential of NPD1 as a therapeutic target for managing chronic pain and enhancing pain control mechanisms in neurological conditions [58].

## 3 PHYSIOLOGICAL ROLE AND MECHANISMS OF NEUROPROTECTIN D1

Neuroprotectin D1 has increasingly been recognized for its critical role in the resolution phase of inflammation by downregulating the expression of various inducible pro-inflammatory genes. Given the emerging role of NPD1 in inflammation resolution, quantifying its plasma levels could become a key biomarker for evaluating its *in vivo* production and efficacy in managing inflammation [59, 60].

Several studies highlight the significant challenges associated with quantifying Specialized Pro-resolving Mediators (SPMs), particularly within the protectin class, such as NPD1, in human fluids. Circulating levels of NPD1 in the blood are reported to be markedly below the quantification threshold, even under conditions of severe inflammation or high-dose omega-3 supplementation. This discrepancy between the low detectable levels and the significant biological efficacy of NPD1 raises questions about whether plasma concentrations accurately reflect *in vivo* production. Additionally, NPD1, like many specialized pro-resolving mediators (SPMs), is challenging to handle due to its susceptibility to isomerization into its *E,E,E-isomer* and its instability in light, air, and acidic conditions. The low concentrations may result from the compound's sensitivity to environmental factors, such as light, air, and acidic pH, which can lead to isomerization and loss of stability. Furthermore, NPD1 may be rapidly cleared from its active sites through local and systemic biotransformation, including hepatic metabolism [61]. The biosynthesis of NPD1 is a complex process involving several enzymatic reactions, primarily within neural tissues, underscoring its localized production in response to physiological and pathological stimuli.

As briefly described above, the process starts with docosahexaenoic Acid (DHA), an omega-3 polyunsaturated fatty acid abundant in neuronal membranes. DHA acts as a precursor for various bioactive lipid mediators involved in inflammation and cellular signaling. The initial step in NPD1 synthesis involves the oxygenation of DHA by 15-LOX enzymes, which convert DHA into 17S-hydroperoxy-DHA (17S-HpDHA). This hydroperoxide is then subjected to reduction and cyclization processes facilitated by additional enzymes, including LOXs. These steps culminate in the formation of NPD1, highlighting the critical role of 15-LOX in its biosynthesis. This biosynthetic pathway is tightly regulated and occurs in a cellular context-dependent manner, being induced in response to inflammation, oxidative stress, and cellular injury [27]. This reflects NPD1's protective role in the central nervous system (CNS) and potentially in peripheral tissues, where it helps modulate inflammatory responses and promote tissue repair. Neuroprotectin D1, an endogenous mediator derived from docosahexaenoic acid (DHA), is synthesized both in the brain and by retinal pigment epithelial (RPE) cells originating from neuroepithelium. This mediator plays a critical role in modulating cellular signaling pathways that enhance cell survival. A primary target of NPD1 is the Bcl-2 family of proteins, which are crucial regulators of mitochondrial apoptotic signaling pathways, especially under conditions of oxidative stress.

NeuroprotectinD1 exerts its protective effects by interacting with these proteins, thereby influencing the cellular response to oxidative damage and promoting cell resilience. By modulating the activity and expression of Bcl-2 family members, NPD1 helps counteract apoptotic signals and maintain mitochondrial integrity, thereby supporting cell survival in adverse conditions. This regulatory role underscores NPD1's potential in safeguarding cells against the detrimental effects of oxidative stress and in preserving cellular function and viability [62]. The distribution of NPD1 is influenced by its transport across cellular membranes and its metabolism by enzymes that affect its bioavailability and duration of action. Understanding these processes is crucial for elucidating the systemic effects of NPD1 and its potential as a therapeutic target.

Specialized pro-resolving mediators (SPMs) exhibit a range of common biological functions, such as reducing neutrophil infiltration, modulation of cytokine profiles from proinflammatory to anti-inflammatory, and enhancing macrophage phagocytosis. Despite these shared roles, SPMs have cell-type-specific effects due to variations in receptor expression across different cell types. The majority of SPM actions are mediated through G protein-coupled receptors (GPCRs) [63]. Given that NPD1 is found in tissues at concentrations significantly below the limit of quantification, further research is essential to fully elucidate and identify its receptor [1].

The biological effects of NPD1 are mediated through specific receptors and intracellular signaling pathways, emphasizing its role as a signaling molecule involved in neuroprotection and inflammation resolution. Moreover, NPD1 may be quickly metabolized or removed from its active sites through rapid local and systemic biotransformation processes, which further complicates the measurement of its levels. Despite these challenges, studies have highlighted the interaction of NPD1 with G protein-coupled receptors (GPCRs), such as GPR37, where it is known to increase intracellular calcium levels and enhance macrophage phagocytosis, indicating its role in successful inflammation resolution [64]. Recent studies have identified the parkin-associated endothelin-like receptor (Pael-R), also known as GPR37, as the specific receptor for NPD1 [64] GPR37 is predominantly expressed in oligodendrocytes and astrocytes but not in microglia [64, 65]. These pathways are crucial for cell survival, anti-inflammatory responses, and the modulation of synaptic plasticity.

Recent studies have elucidated the role of GPR37 in disease progression, including its promotion of colorectal cancer by inhibiting ferroptosis through the p38-SCD1 axis and its potential as a prognostic marker in gliomas [66, 67]. Additionally, Robertson *et al*. showed that GPR37 modulates inflammation-induced gastrointestinal dysmotility by regulating enteric reactive gliosis, suggesting its potential as a therapeutic target for both cancer and gastrointestinal disorders [67]. GPR37 is another receptor implicated in NPD1 actions; however, its specific roles and signaling mechanisms in response to NPD1 require further investigation. Upon binding to its receptors, NPD1 initiates intracellular signaling pathways that regulate gene expression, cytokine production, and cellular responses to oxidative stress and inflammation.

Bang *et al*. provide substantial evidence supporting GPR37 as the specific receptor for NPD1. Their findings include the observation that NPD1, similar to prosaposin and TX14, induces calcium fluxes in HEK293 cells transfected with GPR37, while these effects are not reproduced in cells transfected with other SPM receptors [63]. Moreover, among a panel of SPMs, only NPD1 was capable of eliciting calcium responses in GPR37-expressing cells. Also, genetic ablation of GPR37 eliminated the NPD1-induced calcium responses in primary peritoneal macrophages.

Lastly, dot-blot assays confirmed the binding interaction between NPD1 and GPR37 [64]. NPD1-mediated macrophage phagocytosis of apoptotic remnants relies on signaling through the G protein subunit G_i/o_, ERK, and the PI3K/AKT pathway, in contrast to the involvement of cAMP, pCREB, STAT3, and ERK in the pathway mediated by RvD2/DRV2 [68, 69]. Moreover, the absence of GPR37 was associated with an upregulation of proinflammatory cytokines, including IL-1β, and a concomitant downregulation of anti-inflammatory cytokines, such as IL-10 and TGF-β, in macrophage populations. These findings suggest that the activation of GPR37 plays a pivotal role in favoring the M2 macrophage phenotype, which is associated with anti-inflammatory responses and tissue repair, as opposed to the M1 phenotype, which is linked to proinflammatory activities [63].

Neuroprotectin D1 also plays a prominent role in regulating various other cellular pathways essential for cellular homeostasis. These pathways include the nuclear factor kappa B (NF-κB) pathway, which controls pro-inflammatory gene expression by upregulating the expression of c-Rel, and the phosphatidylinositol-3-kinase (PI3K)/Akt pathway, which supports cell survival and neuroprotection [42, 70]. c-Rel is highlighted as an important NF-κB transcription factor involved in immune regulation and inflammation, with conserved structural and functional roles across animal species [71]. Additionally, melatonin production in macrophages and microglia is critical for their transition from an M1-like to an M2-like phenotype [72, 73]. The upregulation of c-Rel by NPD1 may trigger local melatonin synthesis, which, through autocrine and paracrine signaling, helps dampen inflammatory responses [74].

Neurodegenerative diseases are closely linked to aging, with recent studies suggesting that aging may be influenced by a significant decline in pineal melatonin, along with reduced levels of gut microbiome-derived butyrate and bcl2-associated athanogene 1 (BAG-1), which help regulate and restore body functions during the night as it will be crucial to investigate whether the loss of pineal melatonin, gut butyrate, and BAG-1 affects the activation of NPD1/c-Rel and local melatonin production, as NPD1 could play a vital role in the circadian rhythm [75]. Neuroprotectin D1 exerts potent anti-inflammatory effects by inhibiting the activation of microglia and astrocytes, reducing the production of proinflammatory cytokines such as tumor necrosis factor-alpha (TNF-α) and interleukin-1 beta (IL-1β), and promoting the resolution of inflammation. These actions are essential for maintaining CNS homeostasis and protecting neurons from inflammatory damage [52].

Overall, NPD1 emerges as a pivotal mediator in the resolution of inflammation with a complex biosynthetic pathway, a distinctive distribution pattern, and specific receptor mechanisms. Its role in neuroprotection and systemic inflammation makes it a promising candidate for further research and potential therapeutic applications. Understanding the production, distribution, and mechanism of action of NPD1 will enhance our ability to leverage its benefits in treating various inflammatory and neurodegenerative conditions. As research progresses, the elucidation of biosynthesis, distribution, and receptor interactions of NPD1 will be crucial for developing new strategies to modulate its effects and explore its therapeutic potential.

## 4 ROLE OF NPD1 IN THE PATHOPHYSIOLOGY OF NEURODEGENERATIVE DISEASES

The enzymatic derivative of the omega-3 essential fatty acid NPD1 plays a key role as a lipid mediator in resolving inflammation by downregulating the expression of proinflammatory genes and promoting cell survival by negatively modulating the pro-apoptotic proteins, Bcl-2-associated protein x (BAX) and *BCL2* associated agonist of cell death (BAD). Neuronal tissues constantly strive to manage ROS generated as a result of high metabolic activities and an oxygen-rich environment [76]. This disruption in the homeostasis, as a result of uncompensated oxidative stress, triggers the cleavage of DHA by phospholipase A2 and its subsequent lipoxygenation by 15-LOX-1 into NPD1 [2].

A prominent significance of the neuroprotective effects of NPD1 has been observed in Alzheimer's disease, which is a progressive neurodegenerative disorder characterized by worsening cognitive function. Neuroprotection offered by NPD1 is a result of three important regulatory roles: firstly, as an inducer of anti-apoptotic genes *BCL-2*, *BCL-XL,* and *BFL-1/A1,* secondly, as an inhibitor of anti-apoptotic genes *BAX, BAD,* and *BID* and lastly by shifting the processing of β-amyloid precursor protein (βAPP) from an amyloidogenic pathway to a non-amyloidogenic pathway and by decreasing the production of Aβ42 itself.

### 4.1 Role as an Anti-apoptotic Agent

Recent research has shifted the focus from soluble amyloid-beta (Aβ) as the primary toxic agent in AD to higher-order Aβ complexes, such as protofibrils and oligomers, including Aβ-derived diffusible ligands (ADDLs) or the Aβ-oligomers (Aβo) [77]. These complexes are now considered the main contributors to neurotoxicity. Studies suggest that Aβ can induce apoptosis through various pathways. One hypothesis is that Aβ triggers the extrinsic apoptotic pathway by binding to cell death surface receptors like TNFR, leading to caspase activation. An example of this is the ligation of deubiquitinated receptor-interacting protein 1 (RIP1) to the death-inducing signaling (DISC) complex consisting of TNF receptor-associated factor 2 (TRAF2) and TNFR type-1-associated DEATH domain (TRADD), in the absence of BIRC2 and BIRC3, this interaction activates caspase 8 and induces cell death [76]. The anti-apoptotic proteins BIRC2 and BIRC3 ubiquitinate RIP-1 are involved in both caspase-dependent and independent apoptotic pathways and are, in turn, positively regulated by c-REL, a subunit of NF-κB. The translocation of c-REL homodimers into the nucleus results in the activation of BIRC3 promoter activity, thereby ensuring cell survival [76].

Alternatively, some evidence points to the intrinsic apoptotic pathway as being more significant, with intracellular Aβ playing a crucial role [78, 79]. When Aβ accumulates in the endoplasmic reticulum (ER) or endosomes, where it is partially synthesized can initiate apoptosis through mechanisms like the unfolded protein response or ER stress. Additionally, intracellular Aβ might bind to alcohol dehydrogenase in mitochondria, contributing to mitochondrial stress and further promoting apoptotic pathways [80]. This dual role of Aβ in both extracellular and intracellular compartments highlights the complexity of its involvement in AD pathology [81]. In terms of inhibiting apoptotic pathways during oxidative stress, NPD1 plays a role in upregulating the expression of the anti-apoptotic proteins BIRC2 and BIRC3. These proteins are known to inhibit caspase activity by inducing polyubiquitination of caspases, which leads to their subsequent degradation *via* the proteasome [82].

The caspase inhibitory and anti-apoptotic nature of NPD1 is mediated by the ubiquitination of RIP1 by BIRC proteins. However, the upregulation of promoter activity is brought about by oxidative-stress-induced synthesis of NPD1, which is actively involved in the translocation of c-REL homodimers into the nucleus and their subsequent ligation with the BIRC3 promoter to activate the transcription of *birc3* mRNA. c-REL is known to play a key role in promoting cell survival in human islet cells *in vitro* and enables neuroprotection in the face of induced anoxia [83, 84]. Neuroprotectin D1 also plays a role in negatively regulating the intrinsic pathway of apoptosis by dephosphorylating the anti-apoptotic protein Bcl-xL, which allows it to heterodimerize with Bax and thereby become inactivated.

### 4.2 Role as an Anti-amyloidogenic Agent

The early stages of AD involve neuroinflammation, damage to dendritic spines, and impaired synaptic function, which are exacerbated by oxidative stress and ultimately result in dementia. At the cellular level, it involves synaptic damage, the formation of intracellular neurofibrillary tangles, and dysfunction in the processing of beta-amyloid precursor protein (βAPP), leading to excessive production of the amyloid-beta (Aβ42) peptide, a 42 amino acid long peptide. Aβ42 is implicated in promoting neuroinflammation, synaptic toxicity, and cell apoptosis. Moreover, the presence of Aβ42 is linked to oxidative stress, calcium overload, mitochondrial dysfunction, caspase activation, and subsequent cell death [85-87]. Extracellularly, Aβ42 peptides transition from oligomers to aggregates, which are significant components of senile plaques. Aβ42 peptides are formed from β-amyloid precursor protein (βAPP) through successive cleavage by β- and γ-secretases. Alternatively, the enzyme α-secretase, cleaves βAPP to release a soluble form known as sAPPα *via* the non-amyloidogenic or neuroprotective pathway [88]. These developments correspond with reduced levels of DHA in the brain, which is a precursor to NPD1 biosynthesis [62].

Moreover, NPD1 promotes the processing of amyloid precursor protein (APP) along the non-amyloidogenic pathway, leading to a reduction in the release of amyloid-42 (Aβ42), contributing to the resolution of inflammation caused in the neural tissues. Similarly, both *in vivo* and *in vitro* studies indicate that DHA induces a decrease in Aβ42 levels. This shift from the amyloidogenic to the non-amyloidogenic pathway is a result of the mechanism that involves activating α-secretase and inhibiting β-secretase through the activation of peroxisome proliferator-activated receptor-γ (PPAR-γ) [32]. The regulatory role of NPD1 in impact on amyloid precursor protein (APP) processing as well as non-amyloidogenic pathway, decreasing amyloid-β42 (Aβ42) levels, and resolving neural inflammation, thereby elucidating its neuroprotective mechanisms has been depicted in Fig. (**2**). PPAR-γ is a key anti-inflammatory and Aβ-lowering mediator and hosts a fatty acid binding pocket for polyunsaturated fatty acids, making it a putative receptor for NPD1. PPAR-γ also induces adipocyte differentiation and is involved in lipoprotein metabolism and adipogenesis [89, 90].

Available studies report that the activation of PPAR-γ by NPD1 occurs in a dose-dependent manner observed in the differentiation of primary human adipocytes in an adipogenesis assay carried out by Bazan *et al*. [32]. Conversely, DHA displayed very little adipogenic activity, suggesting that the action of NPD1 is mediated by PPAR-γ rather than by DHA. Additionally, to determine the role of PPAR-γ in the processing of βAPP, βAPP- overexpressing human neuronal-glial (HNG) cells were transfected with PPAR-γ agonists, rosiglitazone and a prominent decrease in the levels of Aβ42 was observed. However, the synthetic PPAR-γ antagonist reversed this reduction in Aβ42 levels, underscoring the key role played by PPAR-γ in the regulation of Aβ42 levels by NPD1 [32]. The two significant β-secretase cleavage products, Aβ42 and CTFβ, were substantially downregulated upon the addition of NPD1, indicating that PPAR-γ is involved in regulation *via* the β-secretase pathway rather than the alpha-secretase pathway since sAPPα and CTFα, the α-secretase cleavage products, remained unchanged. This finding was further supported by a decrease in the expression levels of β-secretase-1 (BACE-1) and an upregulation in the expression of α-secretase disintegrin and metalloproteinase 10 (ADAM-10).

### 4.3 Role as an Anti-inflammatory Agent

Neuroprotectin D1, classified as an SPM, plays a role in limiting the entry of immune cells into the site of inflammation, downregulating proinflammatory mediators, and finally promoting the clearance of the immunogen, thereby contributing to tissue repair [91, 92]. Previous studies have also established our understanding of the anti-inflammatory role executed by SPMs in a series of mouse models of enteritis, peritonitis, retinopathy, sepsis, and inflammatory pain [93, 94]. Previously, the classical perspective disregarded the role of inflammation in neuropathogenesis, attributing it to the brain's “immunologic privilege.” However, advancements in technology have revealed the significant involvement of inflammatory mediators in AD.

Neurodegeneration, as a cumulative aftermath of inflammatory responses to Aβ42, plays an important role in AD pathology. When exposed to the highly immunogenic Aβ42, activated microglia and astrocytes are stimulated to produce proinflammatory cytokines, which in turn induce neuronal cells to produce Aβ42 precursor protein (APP) and positively regulate the amyloidogenic processing of APP, leading to a surge in the physiological levels of Aβ42. Prior studies on AD have also demonstrated an increase in molecules involved in innate immunity and inflammation, such as IL-1, apart from the presence of activated microglia within and around senile plaques [95, 96]. The microenvironment of damaged tissues and cellular debris further exacerbates the neurodegenerative inflammation and reinforces the pathogenesis cycle.

A complex array of inflammatory subsystems, governed by the chemokines, cytokines, complement, and acute phase proteins, becomes active in AD, each characterized by numerous amplifying and dampening loops, as well as interactions with other subsystems. These interactions resemble a web, where different inflammatory pathways can influence each other, potentially leading one set of mediators to induce others. Fig. (**3**) illustrates the complex inflammatory processes in neurodegeneration, where amyloid-β42 (Aβ42) triggers the activation of microglia and astrocytes, leading to the production of proinflammatory cytokines. I*n vitro* studies have demonstrated that β-pleated fibrillar Aβ and, more recently, tau-containing neurofibrillary tangles directly activate the classical complement pathway. Specifically, a sequence of 13-15 amino acids on the collagen-like tail of the human C1q A chain contains five cationic side chains that bind in a charge-dependent manner to anionic side chains on the N-terminus of human Aβ [97].

Neurodegeneration can expose DNA and neurofilaments to the extracellular environment. DNA has been shown to activate C1 by binding to the same site on the C1q A chain used by Aβ, tau, and other activators, independent of antibodies. Neurofilaments, oligodendrocyte myelin glycoprotein, and other myelin-derived proteins also activate the classical complement pathway *in vitro*. Therefore, an increase in their presence can activate the classical complement pathway, leading to the formation of the membrane attack complex (MAC) and subsequent lysis of healthy cells due to the bystander effect [88]. Studies indicate that microglia and astrocytes are primary endogenous sources of complement proteins in an AD brain, further substantiating the role these cells play in activating complement pathways and consequently contributing to neurodegeneration in AD patients [80, 98-101].

Almost all cytokines and chemokines investigated in AD, including IL-1β, IL-6, TNF-α, IL-8, transforming growth factor-beta (TGF-β), and macrophage inflammatory protein-1α (MIP-1α), exhibit increased expression levels in AD compared to samples from individuals without the disease. IL-1β, particularly, stimulates both the synthesis [102] and processing of APP, thereby potentially enhancing amyloid production and deposition in plaques. Additionally, the secreted form of APP (sAPP) reciprocally activates microglia and triggers increased expression of IL-1β, thereby forming a positive feedback loop, contributing to the formation of plaques [36]. Another cytokine, IL-6, in broader contexts, is often regarded as a harmful, pro-inflammatory cytokine that stimulates the production of acute phase proteins, acts as a potent pyrogen, and enhances vascular permeability, lymphocyte activation, and antibody synthesis [88].

Tumor necrosis factor-alpha exhibits a dualistic function mediated by its interaction with two distinct receptors: the p55 TNF receptor (type I TNFR) and the p75 TNF receptor (type II TNFR) [103, 104]. Type I TNFR is implicated in pro-apoptotic signaling pathways, while type II TNFR is involved in cellular survival mechanisms [105, 106]. However, TNF- α shows highly potent cytopathic effects in several studies carried out on human cortical neurons [105, 107, 108] and is associated with severe inflammation and encephalopathy. Inflammation is also partly regulated by COXs, which synthesize PGs *via* the arachidonate cascade.

Studies on glial cultures suggest that PGs, especially prostaglandin E2 (PGE2), influence the production of various inflammation-related molecules such as IL-6, chemokines, and APP. While further research is necessary, it is plausible that the production of chemokines associated with plaque formation contributes to the recruitment and accumulation of astrocytes and microglia within senile plaques [88]. The intricate interplay of glial cells, cytokines, chemokines, and complement proteins exacerbates inflammation in cerebral tissues, crucially contributing to AD pathogenesis. This dysregulation underscores the disease's progression, highlighting inflammation as a pivotal factor in its development, which is effectively countered by the anti-inflammatory effect of NPD1.

### 4.4 Role as an Antioxidant Agent

Apart from this, there is a growing interest in the role played by the production of free radicals in the development and progression of neuropathology. The presence of malondialdehyde, 8-hydroxy-deoxyguanosine, 4-hydroxy-nonenal, and nitrotyrosine, along with an increase in lipid peroxidation in the AD brain, indicates oxidative stress-mediated damage to neuronal tissues at the molecular level [88, 107, 109, 110]. As a direct consequence of the generation of free radicals, there is an upregulation in the expression of NF-κB mediated transcription of several pro-inflammatory and pro-apoptotic genes, which undermines neuronal integrity. The components of nicotinamide adenine dinucleotide phosphate (NADPH) oxidase complex, present in the activated microglia in an inactive form, are assembled to activate the complex in response to the introduction of Aβ peptides which results in a burst of superoxide radicals [88, 111].

The production of nitric oxide (NO), another ROS, is tremendously elevated in AD brains. However, the specific origin of NO in the AD brain has not been definitively identified. Several studies suggest that NO may be produced by various cell types, including microglia, astrocytes, neurons, and endothelial cells within the brain [112, 113]. Myeloperoxidase (MPO) enzyme found in microglial cells neighbouring Aβ42 plaques catalyses the production of hypochlorous acid from hydrogen peroxide and chloride, which in turn reacts with other molecules to generate more ROS [114]. Neuronal death, caused by oxidative stress, is yet another significant cause of the progression of neuropathology in AD.

Conclusively, NPD1 plays a pivotal role in restoring homeostasis in neuronal tissues by halting apoptosis, resolving inflammation, and shifting the amyloidogenic processing of Aβ42 to the non -amyloidogenic pathway while positively modulating cell proliferation, as has been depicted in Fig. (**4**). As has been discussed earlier, NPD1 plays a crucial role in redirecting the processing of APP from the amyloidogenic to the non-amyloidogenic processing pathway by upregulating the expression of α-secretase and inhibiting β-secretase, which is primarily involved in the synthesis of Aβ42, mediated by PPAR-γ activation. Like other mediators, Aβ42 is highly immunogenic and elevates the levels of pro-inflammatory cytokines like IL-1β. As NPD1 reduces Aβ42 levels, it significantly decreases the oxidative stress caused by the accumulation of amyloid plaques.

## 5 PHARMACOLOGICAL CONGENERS OF NPD1 AND THEIR EFFECT

Specialized pro-resolving mediators (SPMs); are a class of endogenously present chemical mediators involved in the initiation, modulation, and mitigation of inflammation and its subsequent resolution. Restoration of homeostasis is facilitated by the active biosynthesis and metabolism of these mediators; therefore, it is of great interest to explore several other chemical mediators that act as agonists or antagonists to their functionality in maintaining cellular homeostasis. Several reports have identified immediate downstream metabolites or isomers of NPD1, such as 22-hydroxy-NPD1, Protectin DX, aspirin-triggered PD1, and 10-epi-PD1 [14, 24, 115, 116]. These compounds either demonstrate similar efficiency to NPD1 or exhibit even greater effectiveness in resolving inflammation and restoring cellular homeostasis.

Additionally, several other analogs have been reported, most notably the synthetic analog, 3-oxa-PD1_n-3 DPA_, a beta-oxidation-resistant mimic of protectin-D1 n-3 docosapentaenoic acid (PD1_n-3 DPA_), which showed equal potency as PD1 and PD1_n-3 DPA_ in reducing neuropathic pain as well as chronic itch in animal models, thereby retained the potent bioactions of these two SPMs [117]. Furthermore, the first synthetic analog of protectin-D1, named 22-Fluoro-10,17-dihydroxydocosa-4Z,7Z,10R,11E,13E,15Z,17S,19Z-hexanoic acid (22-F-PD1), has also demonstrated to exert its potential as an anti-inflammatory and pro-resolving mediator [118].

### 6 22-HYDROXY-NPD1(10(R),17(S),22-TRIHYDROXYDOCOSA-4Z,7Z,11E,13E,15Z,19Z-HEXAENOIC ACID)

The lipid mediator PD1 has been widely investigated for its potential as a novel anti-inflammatory drug and is currently undergoing clinical trials. Given the growing interest in Specialized Pro-Resolving Mediators (SPMs) for their crucial roles in promoting the resolution of inflammation, significant research has been carried out to elucidate their downstream metabolic pathways. These studies aim to provide a deeper understanding of how SPMs are metabolized and how their metabolic products contribute to their effectiveness in resolving inflammatory responses.

Initial research conducted in 2003 focused on the metabolism of PD1, leading to the identification of trace amounts of a 10,17,22-trihydroxy C22 polyunsaturated fatty acid. The structure of this compound was elucidated through a combination of biosynthetic analysis and LC-MS/MS data [12]. The organic synthesis of 22-hydroxy NPD1 was done by Hansen *et al.* to understand the pharmacological significance of this metabolite in mouse models [15].

A critical aspect of inflammation resolution involves inhibiting the infiltration of PMNs as they migrate into affected tissues during the acute phase of inflammation, also reducing pro-inflammatory mediators’ actions that are central to the function of specialized pro-resolving mediators (SPMs). Analysis of peritoneal inflammatory exudates in a murine model revealed that PMN infiltration was inhibited to a level comparable to that achieved by NPD1. This 22-hydroxylated-metabolite of NPD1 also demonstrated a significant reduction in the cellular levels of pro-inflammatory lipid mediators like leukotriene-B4 (LTB_4_), thromboxane-B2 (TXB_2_) and prostaglandin-F2α(PGF_2α_). While ω-oxidation generally leads to a dramatic reduction-approximately 100-fold in the activity of many bioactive fatty acid metabolites such as leukotriene B4, 5-HETE, and 5-oxo-eicosatetraenoic acid (5-oxo-ETE), the ω-oxidized form of protectin D1 (PD1) exhibits notable activity.

As discussed above, this oxidized derivative displays strong anti-inflammatory and pro-resolving effects, including the inhibition of PMN chemotaxis in both *in vivo* and *in vitro* settings. Moreover, it reduces levels of pro-inflammatory mediators in animal model inflammatory exudates to an extent comparable to that of PD1 itself [118, 119]. Under normal physiological conditions, protectin D1 undergoes ω-oxidation catalysed by cytochrome P450 (CYP) enzymes, resulting in the formation of its monohydroxylated derivative [116, 119].

## 7 PROTECTIN DX (10(S),17(S)-DIHYDROXYDOCOSA-4Z,7Z,11E,13Z,15E,19Z-HEXAENOIC ACID)

10*S*,17*S*-dihydroxy-4*Z*,7*Z*,11*E*,13*Z*,15*E*,19*Z*-docosahex-aenoic acid or protectin DX (PDX), is the 13*Z*,15*E*,19*Z* isomer of NPD1, which has the 13*E*,15*Z*,19*Z* double bond configuration. In conclusion, PD1 and PDX differ in two key aspects: the geometry of the double bonds in the conjugated triene and the configuration of carbon 10. Specifically, PDX has an E,Z,E configuration for its conjugated triene, while PD1 features an E,E,Z configuration.

Additionally, the configuration at carbon 10 is S in PDX and R in PD1 [120]. Although PD1 and PDX are structural isomers, they exhibit similar functional properties, with PDX showing some additional activities, including its role in inhibiting platelet aggregation induced by collagen or arachidonic acid at submicromolar concentrations by blocking COX-1. It also counteracts thromboxane A2-induced platelet aggregation. PDX belongs to a classified group of oxygenated trienes of polyunsaturated fatty acids, known as poxytrin, that are characterized by their distinct E,Z,E-conjugated triene structure flanked by two hydroxyl groups [115].

Furthermore, PDX is a potent anti-aggregatory and anti-inflammatory agent as it plays a significant role in inhibiting both cyclooxygenase-1 and cyclooxygenase-2, thereby inhibiting the synthesis of pro-inflammatory PGs. Additionally, PDX inhibits NADPH oxidase (NOX) and reduces the release of myeloperoxidase (MPO) from neutrophils, thereby decreasing the production of ROS [121].

## 8 ASPIRIN-TRIGGERED PD1(10(R),17(R)-DIHYDR-OXY-DOCOSA-4Z,7Z,11E,13E,15Z,19Z-HEXAENOIC ACID

10*R*,17*R*-dihydroxy-4*Z*,7*Z*,11*E*,13*E*,15*Z*,19*Z*-docosahe-xaenoic acid, commonly referred to as Aspirin-triggered PD1 (AT-PD1) or 17-epi-PD1 and is a 17R-hydroxyl diastereomer of PD1 since it has a 17R rather than 17S hydroxy residue. SPMs are typically synthesized through two endogenous pathways, namely the aspirin-dependent pathway and the other being LOX-mediated pathway [23, 53].

Neuroprotectin D1 is no exception to this and can be synthesized *via* both the aspirin-dependent COX-2 route, which generally synthesizes 17R dominant species, and the LOX-mediated pathway, which generates 17S-dominant mediators. Acetylated by aspirin, COX-2 catalyses the conversion of DHA to 17R-hydroperoxy-containing-DHA-derived metabolite (17R-HpDHA). This metabolite is further converted to both 17R-aspirin-triggered-D-series resolvins (AT-RvD) or aspirin-triggered-D-series protectins (AT-PD) through the formation of a 16R, 17R-epoxide intermediate [24].

AT-PD1 demonstrated a steep reduction in PMN infiltration and significantly reduced the transendothelial migration of PMNs by LTB_4_. Additionally, AT-PD1 enhanced the removal of apoptotic PMNs by macrophages in a process known as efferocytosis [23]. Serhan *et al*. have also reported the synthesis of this epimer using a stereochemically controlled chemical process, demonstrating the potential of this isomer of PD1 in resolving inflammation [4].

## 9 10-EPI-PD1 (10*S*,17*S*-DIHYDROXY-4*Z*,7*Z*,11*E*,13*E*, 15*Z*,19*Z*-DOCOSAHEXAENOIC ACID)

10*S*,17*S*-Dihydroxy-4*Z*,7*Z*,11*E*,13*E*,15*Z*,19*Z*-docosahexaenoic acid, commonly termed as 10-Epi-PD1, is the 10*S*-hydroxy diastereomer of PD1, which contains a 10*R*-hydroxy residue. Reportedly, it was first synthesized by Petasis *et al.* in 2006 to understand the stereochemistry of PD1 and its isomers [14, 119]. The primary distinction between **10-Epi-PD1** and **PD1** lies in the chirality of the carbon at position 10; **10-Epi-PD1** has the S configuration, while **PD1** has the R configuration. Aside from this chirality difference, both compounds share identical triene geometry.

Furthermore, their physiological concentrations differ, as **10-Epi-PD1** is found in cells only in trace amounts, whereas **PD1** is present in more substantial quantities. This observation raises intriguing questions about the enzyme responsible for converting the proposed epoxy intermediate into PD1 [12]. Specifically, the enzyme exhibits a high degree of selectivity in incorporating water-derived alcohol at carbon-10, resulting predominantly in the 10R configuration of PD1, while only trace amounts of the 10S configuration, as seen in 10-Epi-PD1, are produced. This selectivity in enzyme function warrants further investigation to understand its underlying mechanisms better. 10-Epi-PD1 was detected in only a small amount in human PMN extracts, yet it was more potent than PD1 or PDX in blocking the inflammatory response to zymosan A-induced murine acute peritonitis, as it drastically reduced PMN infiltration [119]. The methyl ester of this compound also proved to be a potent regulator of PMN infiltration [14]. Recently, Durand *et al*. have reported a more stereocontrolled and efficient process for the synthesis of this epimer [119].

## CONCLUSION

Neuroprotectin D1 plays a crucial role in resolving inflammation and oxidative stress, which contributes to the neuroprotective effects. NPD1 functions primarily through specific receptors such as GPR32 (ChemR23) and GPR37. The activation of GPR32 triggers downstream signaling pathways, including Akt and ERK, which are essential for cell survival and anti-inflammatory responses. The actions of NPD1 include suppressing pro-inflammatory cytokines like IL-1β, TNF-α, and IL-6, as well as inhibiting the NF-κB pathway, which is crucial for pro-inflammatory gene expression. Neuroprotectin D1 also modulates microRNAs linked to neuroinflammation and neurodegeneration, such as miRNA-125b. In neurodegenerative diseases like AD, NPD1 reduces Aβ42 levels, mitigating inflammatory responses and promoting the resolution of neuroinflammation. It regulates apoptosis by enhancing anti-apoptotic proteins and inhibiting pro-apoptotic pathways. The role of NPD1 in maintaining mitochondrial function and regulating Bcl-2 family proteins facilitates neuronal survival under oxidative stress. Neuroprotectin D1 also plays a role in negatively regulating the intrinsic pathway of apoptosis by dephosphorylating the anti-apoptotic protein Bcl-xL, which allows it to heterodimerize with Bax and thereby become inactivated. As discussed earlier, NPD1 plays a crucial role in redirecting the processing of APP from the amyloidogenic to the non-amyloidogenic pathway by upregulating the expression of α-secretase and inhibiting β-secretase, which is primarily involved in the synthesis of Aβ42, mediated by PPAR-γ activation.

Additionally, NPD1 enhances the phagocytic activity of microglia, facilitating the clearance of cellular debris and apoptotic cells, which is vital for inflammation resolution and tissue repair. It also stabilizes the blood-brain barrier (BBB), reducing vascular permeability and preventing immune cell infiltration. Additionally, NPD1’s ability to modulate pain is linked to its inhibition of nociceptor channels such as TRPA1 and TRPV1, reducing neuroinflammatory pain by affecting PKA and ERK signaling pathways. This dual action on nociceptive receptors and neuroinflammatory pathways underscores the potential of NPD1 as a therapeutic target for chronic pain management. Research into NPD1 analogs and metabolites, such as 22-hydroxy-NPD1 and synthetic derivatives like 3-oxa-PD1n-3 DPA and 22-F-PD1, indicates their potential to mimic or enhance NPD1’s effects. These compounds may offer new therapeutic avenues for treating inflammatory and neurodegenerative conditions. Despite the development of analogues, measuring NPD1 levels in plasma is challenging due to its low concentrations, even in severe inflammatory conditions or with high-dose omega-3 supplementation. This discrepancy suggests that plasma levels may not accurately reflect endogenous NPD1 production. The instability of NPD1, including its tendency to isomerize and undergo rapid biotransformation, further complicates its quantification. However, the available literature is suggestive of NPD1 as a pivotal mediator in inflammation resolution and neuroprotection. Understanding the mechanism of action will enhance its potential application in managing inflammatory and neurodegenerative diseases.

## FUTURE DIRECTIONS

As highlighted in the review, NPD1's potential as a powerful anti-inflammatory, antioxidant, anti-amyloidogenic, and anti-apoptotic agent makes it a promising candidate for therapeutic applications. However, future research on the identification and comprehensive characterization of NPD1 and its specific receptors, particularly GPR37 NPD1, offers a promising horizon for therapeutic targeting and can be useful for potential health benefits. A molecular understanding of the receptor-ligand interactions between NPD1 and GPR37 will be instrumental in defining the precise role of NPD1 in cellular homeostasis and in identifying potential therapeutic targets.

Low concentrations of NPD1 pose a challenge for its quantification. Developing more sensitive and robust analytical techniques, such as advanced mass spectrometry or highly specific immunoassays, may enable more accurate measurement of NPD1 levels in tissues and bodily fluids for understanding not only the physiological and pathological roles of NPD1 but also for evaluating its neuroprotective and anti-inflammatory efficacy in a therapeutic context and monitoring its pharmacokinetics in neurodegenerative diseases. A deeper investigation into the biochemical pathways modulated by NPD1 is warranted to understand how NPD1 affects oxidative stress, apoptosis, and cellular repair mechanisms that will elucidate its broader biological roles and therapeutic potential. Detailed studies on how NPD1 interacts with other SPMs could reveal complex regulatory networks involved in inflammation resolution and tissue repair. An enhanced synergistic interaction of NPD1 and RvD1 in promoting neuronal homeostasis has been demonstrated. Specifically, the synergistic interplay between the SPMs facilitates robust resolution of neuroinflammation and supports the preservation of neuronal function and integrity. Investigating various formulations and delivery methods can also offer the practical application of NPD1 in clinical settings. Comprehensive studies are needed to evaluate potential adverse effects associated with NPD1 treatment, ensuring that its therapeutic benefits outweigh any associated risks. By addressing these avenues, we can deepen our understanding on the role of NPD1 in health and disease, optimizing its potential as a therapeutic agent and contributing to the development of novel treatment strategies for inflammatory and neurodegenerative disorders. This multi-faceted approach will not only enhance our fundamental knowledge of NPD1 but also translate into practical and impactful clinical applications, ultimately improving patient outcomes across a spectrum of conditions.

## Figures and Tables

**Fig. (1):**
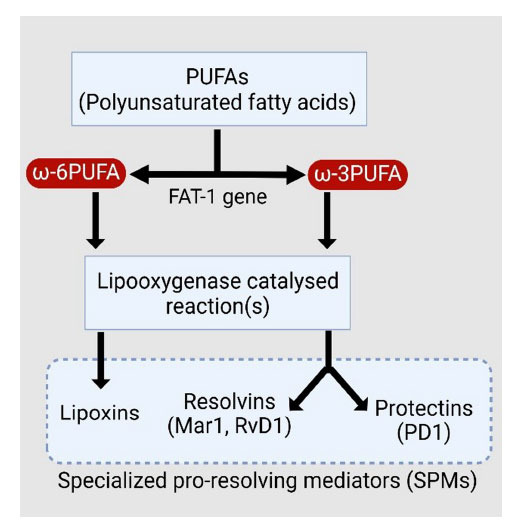
Classification of various specialized pro-resolving mediators (SPMs).

**Fig. (2):**
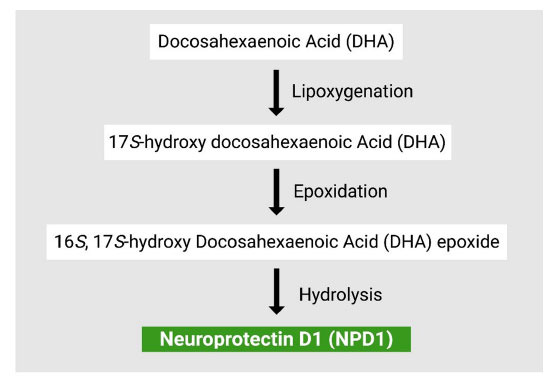
Biosynthesis of Neuroprotectin D1 (NPD1) from Docosahexaenoic acid (DHA).

**Fig. (3):**
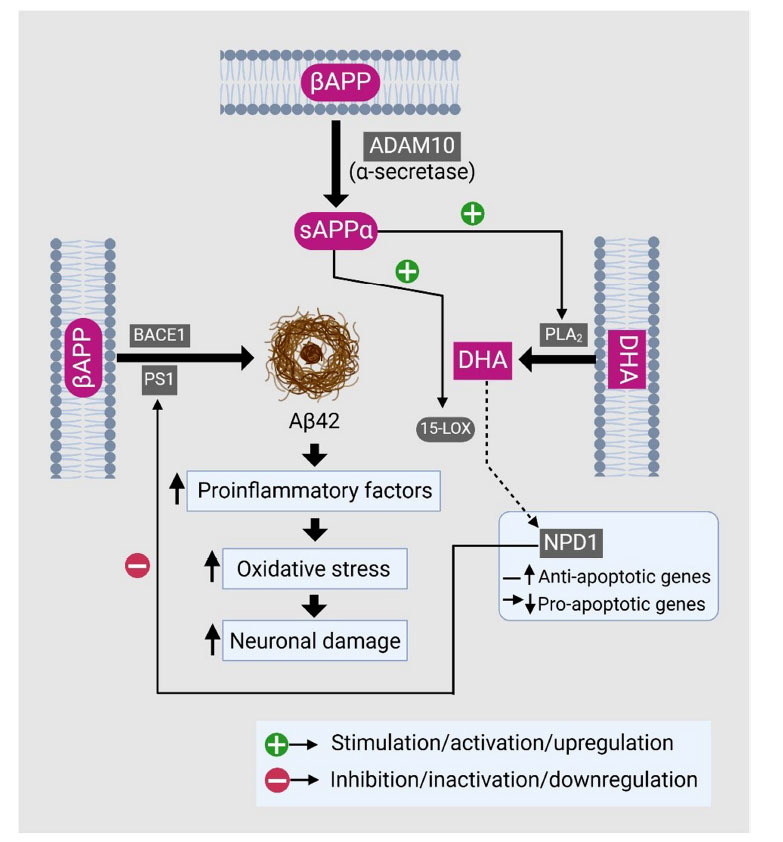
Role of Neuroprotectin D1 (NPD1) in regulating the complex amyloidogenic and apoptotic pathways.

**Fig. (4):**
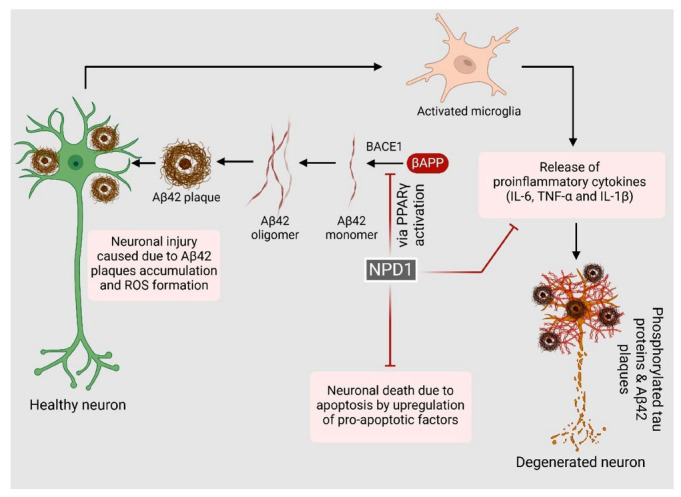
Role of Neuroprotectin D1 in neurodegeneration. Aβ42 activates microglia, resulting in elevated levels of proinflammatory cytokines that exacerbate neuronal amyloid precursor protein (APP) processing and Aβ production, thus establishing a self-perpetuating cycle of neurodegeneration. This inflammatory response is further aggravated by tissue damage and cellular debris. Neuroprotectin D1 exerts neuroprotective effects through several mechanisms: modulating APP processing to reduce amyloidogenesis and enhancing Aβ clearance by microglia; shifting microglial profile from a proinflammatory to an anti-inflammatory phenotype, which decreases proinflammatory cytokine production; and attenuating the expression of pro-apoptotic factors, thereby promoting neuronal survival.

## LIST OF ABBREVIATIONS

AD: Alzheimer’s Disease
ADDLs: Aβ-derived Diffusible Ligands
APP: Amyloid Precursor Protein
Aβ: Amyloid-beta
BBB: Blood-brain Barrier
CNS: Central Nervous System
COXs: Cyclooxygenases
DHA: Docosahexaenoic Acid
DRG: Dorsal Root Ganglion
GPCRs: G Protein-coupled Receptors
IL-1β: Interleukin-1β
IL-6: Interleukin-6
LOX: Lipoxygenase
LTs: Leukotrienes
MAC: Membrane Attack Complex
MPO: Myeloperoxidase
NF-κB: Nuclear Factor-kappa B
NO: Nitric Oxide
NPD1: Neuroprotectin D1
PBMC: Peripheral Blood Mononuclear Cells
PD: Parkinson's Disease
PD1: Protectin D1
PGs: Prostaglandins
PMNs: Polymorphonuclear Neutrophils
RPE: Retinal Pigment Epithelial
SPMs: Specialized Pro-resolving Mediators
TJ: Tight Junction
TNF-α: Tumor Necrosis Factor-alpha
TXs: Thromboxanes
VDR: Vitamin D3 Receptor
